# Azelaic Acid: A Bio-Based Building Block for Biodegradable Polymers

**DOI:** 10.3390/polym13234091

**Published:** 2021-11-24

**Authors:** Anamaria Todea, Caterina Deganutti, Mariachiara Spennato, Fioretta Asaro, Guglielmo Zingone, Tiziana Milizia, Lucia Gardossi

**Affiliations:** 1 Department of Chemical and Pharmaceutical Sciences, University of Trieste, Via L. Giorgieri 1, 34127 Trieste, Italy; atodea@units.it (A.T.); cdeganutti@units.it (C.D.); mariachiara.spennato@phd.units.it (M.S.); fasaro@units.it (F.A.); zingone@units.it (G.Z.); 2 Novamont S.p.A., Via G. Fauser 8, 28100 Novara, Italy; tiziana.milizia@novamont.com

**Keywords:** azelaic acid, bio-based monomers, bio-based polymers, biodegradability, biocompostability

## Abstract

Azelaic acid is a dicarboxylic acid containing nine C atoms, industrially obtained from oleic acid. Besides its important properties and pharmacological applications, as an individual compound, azelaic acid has proved to be a valuable bio-based monomer for the synthesis of biodegradable and sustainable polymers, plasticizers and lubricants. This review discusses the studies and the state of the art in the field of the production of azelaic acid from oleic acid, the chemical and enzymatic synthesis of bio-based oligo and polyester and their properties, including biodegradability and biocompostability.

## 1. Introduction

The production of fine chemicals, new materials and products from renewable feedstocks represents a continuous challenge. Several procedures have been reported in the literature or patented in the last decade for the main biomass components: carbohydrates (75%), lignins (20%), fats and oils (5%) [1]. Regarding oleochemical developments, the oxidative cleavage of unsaturated fatty acids to produce dicarboxylic acids, hydroxy acids, and amino acids has received great attention in the last decade [2]. Two main oleochemical products obtained by the cleavage of unsaturated fatty acids are sebacic acid and azelaic acid.

Azelaic acid (AzA) is a naturally occurring saturated nine carbon atom dicarboxylic acid found in whole grains, wheat, rye and barley [2], first detected in rancid fats. It can be formed endogenously from substrates such as longer-chain dicarboxylic acids and processes like the metabolism of oleic acid, and ψ-oxidation of monocarboxylic acids. The azelaic acid market is predicted to reach USD 160 million by 2023 and the applications include pharmacological ingredients, polymers, plastics, lubricants and materials for electronics [3]. The aim of the present review is to highlight the potential of azelaic acid as powerful building block for the synthesis of bio-based and biodegradable polymers, with a special emphasis on the green synthetic routes, embracing both chemical and enzymatic methods.

## 2. Azelaic Acid: A Bio-Based Monomer with Pharmacological Properties

The pharmacological applications of azelaic acid have been studied since the 1980s [4] and azelaic acid has been approved by both the FDA (US Food and Drug Administration) and by the EMA (European Medicines Agency) for different external uses. Formulations of AzA (15–20% *w*/*w*) are used in the treatment of inflammatory acne vulgaris with medium to moderate severity [5]. 

Moreover, it is widely used in the treatment of skin pigmentation [6,7] and melasma [8]. Concerning the mechanism of action, azelaic acid exerts an inhibitory effect against tyrosinase, a key enzyme for the synthesis of melanin and in this sense, it is active, above all, at the level of hyperactive melanocytes, while sparing normal ones. Kinetic studies have revealed that the inhibitory action is linked to the presence of the two carboxylic groups at the end of the carbon chain [9]. In particular, the mechanism sees an acid-base interaction between the two functional groups of azelaic acid and the residues of histidines present in the catalytic site, linked to copper [9,10].

However, AzA may also exert its activity against tyrosinase indirectly by inhibiting the interaction with thioredoxin reductase associated with the plasma membrane [11]. The dithiol form of thioredoxin is a powerful cellular reducing agent involved in defense reactions against oxidative stress mainly due to photochemical reactions between ultraviolet rays (UV) and oxygen present at the molecular level. Thioredoxin reductase catalyzes the disulfide reduction of oxidized thioredoxin by NADPH, leading to the formation of two thiol groups that bind the active site of the tyrosinase enzyme [12]. When azelaic acid inhibits the reduction of extracellular chemical species, electrons flow in the direction of oxidized thioredoxin to increase the intracellular concentration of reduced thioredoxin that acts as a potent inhibitor of tyrosinase thus preventing melanin biosynthesis. It has been seen that the catalysis of the melanogenesis enzyme is reduced by 58% in the presence of reduced thioredoxin, compared to the activity performed when all the thioredoxin is in an oxidized form.

Furthermore, the thioredoxin reductase/thioredoxin system is shown to be a principal electron donor for the ribonucleotide reductases which regulate DNA synthesis [13]. Consequently, the inhibitory action on the enzyme could be exploited as a therapeutic benefit even in diseases related to tumor proliferation and to colonization by pathogens. In fact, it has been found that thioredoxin reductase and thioredoxin are overexpressed in many aggressive tumors, where they participate in carcinogenesis, tumor progression and drug resistance. In this case, thioredoxin is involved not only in the activation of ribonuocleotide reductase, but also in promoting many other biochemical pathways, very often not desired [14]. It follows that the inhibition of thioredoxin reductase would lead to the advantage of blocking all the activities mediated by the thioredoxin [15].

The inhibition of DNA synthesis gives azelaic acid bacteriostatic properties, useful in treating diseases with the presence of bacteria as elements of etiopathogenesis. In particular, it has been shown that the active ingredient has clinical efficacy in acne therapy and that the inhibition of thioredoxin reductase, located in the bacterial cytosol, is one of the mechanisms of action of AzA [11]. Formulations of azelaic acid (20% *w*/*w*), applied twice a day, cause a reduction in intrafollicular microbial populations, with a 2500-fold reduction of the *S. epidermidis* microorganism after 2 months, together with a 97.7% decrease in the concentration of *P. acnes* [5]. In addition, the inhibition of mitochondrial enzymes by azelaic acid, determines the blocking of cellular respiration, with consequent cytostatic effects, or cytotoxic if at higher concentrations, the inhibition being dose dependent [16]. Nevertheless, the total safety of administering the drug in humans has been highlighted several times, since in vitro and in vivo it does not bring toxic effects to cells not affected by pathologies. As a result, dicarboxylic acid can be exploited when it is necessary to intervene against abnormal cells. This is the case of cancer cells, which are more sensitive to antimitochondrial agents and internalize azelaic acid within them in quantities three times higher than normal cells [17]. Therefore, preferential cytotoxic and antiproliferative effects are obtained on tumor cells, through the blocking action of cellular respiration and the interruption of DNA synthesis, by inhibiting the enzymes mentioned so far.

In the presence of the acne pathology, the keratinocytes hyperproliferate and differentiate in an anomalous way, and in addition to this the desquamation process is also altered. Consequently, there is an accumulation of dead cells that induces the formation of primary lesions, defined as microcomedones. The set of various factors can subsequently cause microscopic lesions to evolve into inflammatory or noninflammatory comedones, typical of acne [18]. Various studies have shown that azelaic acid modulates epidermal differentiation in vivo and that it has a marked antiproliferative cytostatic effect on keratinocyte cultures in vitro. This activity is primarily the consequence of the indirect inhibitory action on ribonucleotide reductase and of the competitive inhibitory action on the fundamental enzymes of the mitochondrial respiratory chain. However, the differentiation of keratinocytes is mainly influenced by the reduction of protein synthesis of keratin precursors. In clinical practice, the reduction in the number of keratinocytes in the skin surface is linked to a lower formation of comedones, followed by a marked improvement in acne-affected skin. Therefore, after long-term exposure to the active ingredient, protein distribution returns to normal, with advantageous results in therapy [19].

The AzA anti-inflammatory response [20,21,22] and activity [23,24] were demonstrated as well. It has been shown that the molecule is efficient in inhibiting in vitro the oxidation of aromatic compounds and the lipoperoxidation of arachidonic acid (C 20: 4, n 6), which are processes induced by the presence of hydroxyl radicals (HO∙). These studies have shown that azelaic acid acts as a “free radical scavenger”, in other words, it mitigates the toxic effect of reactive oxygen species (ROS), which are powerful chemical mediators of the inflammatory response [25]. It has also been shown that azelaic acid can prevent the production of ROS, such as superoxide anion (O^2−^) and hydroxyl radical (HO^−^), generated by human neutrophils, without interfering with chemotaxis and phagocytosis. A significant decrease in oxidative tissue damage at the inflammation site is thus obtained, both for the direct mechanism on free radicals and for the inhibitory action on the production of new radicals, mediated by neutrophils.

Arresting the development of ROS represents in itself a possible strategy in the control of diseases such rosacea, another very common pathology that affects the skin, and in particular the facial area. Although the pathogenesis of rosacea has not yet been fully understood, free radical damage has been found to worsen the clinical condition [26]. Nevertheless, it is the keratinocytes that play a decisive role in promoting the inflammatory cascade, which favors the onset of skin diseases. In fact, keratinocytes lead to the production of immune signals and the secretion of cytokines, in response to various promoter factors, including UV rays [22].

## 3. Azelaic Acid Synthetic Routes

An ozonolysis method for industrial production of bifunctional monomers, such as azelaic acid, has been applied by some oleochemical companies, like Emery, Croda Sipo and more recently P2 science. This process presents some disadvantages such as high energy and technologic demand to produce ozone and some potential risks associated with ozone utilization [27]. The ozonolysis route involves firstly a primary ozonide of oleic acid and ozone via 1,3 cycloaddition. Secondly, the resulting 1,2,4-trioxolane is oxidized to carboxylic acids under oxidative reaction conditions [28].

The application of hydrogen peroxide as an oxidant has been industrialized in Porto Torres by Matrica (“Novamont S.p.A.-Chimica Vivente per la Qualità della Vita”, 2019) by building up a plant capable of producing azelaic and pelargonic acid by different sources using 25,000–30,000 tons/year of vegetable oils [27]. The patented processes of Novamont S.p.A. related to the valorization of vegetable oils directly into different carboxylic acids include three main steps: (i) the triglycerides olefinic double bong oxidation by using an aqueous solution of hydrogen peroxide to obtain an intermediate compound containing vicinal diols, (ii) a second oxidation step of the two hydroxyl groups of the vicinal diol to carboxylic groups using a compound containing oxygen and a catalyst capable of catalyzing the oxidation and (iii) a hydrolysis reaction of acidic triglycerides after separation of the monocarboxylic acids. By this method, azelaic acid or brassylic acid can be obtained with yields of up to 80% [29,30].

**Figure 1:**
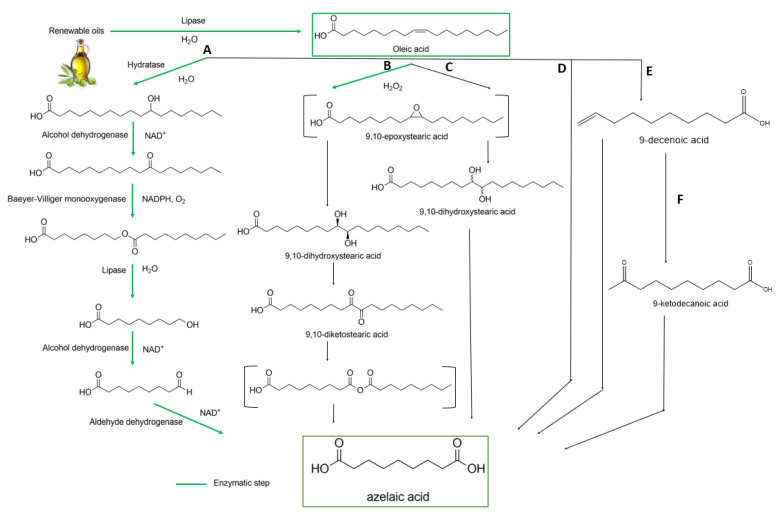
The main routes reported for synthesis of azelaic acid starting from oleic acid: **A**—enzymatic route [39], **B**—chemo-enzymatic route [2], **C**—two step route with epoxide [34], **D**—direct cleavage, **E**—two step pathway with metathesis of oleic acid, **F**—three step route [28].

Different strategies for the improvement of AzA production have been reported in the literature and they were focused on one step ozonolysis, on the optimization of the oxidation reaction from the second step or on the enzymatic and chemo-enzymatic synthesis. Figure 1 illustrates the main routes reported for the synthesis of azelaic acid starting from oleic acid.

An improvement of the second oxidation step of oleic acid was achieved in the 1960s by implementing the in situ formation of performic acid from H_2_O_2_ and HCOOH in combination with ozonolysis to yield AzA up to 95% [31]. Lower yields (71%) were obtained when oxidation was performed using H_2_O_2_, phosphotungstic acid, or tungstic acid as the catalyst precursor and quaternary ammonium salts as phase transfer catalysts [32]. Other oxides containing Mo-, V-, Mn-, Co-, Fe-, and Pb- or salts and tungstic acid were reported with yields of AzA ranging from 70 to 87%. The decomposition of the secondary ozonide product was investigated by using microwaves without a catalyst and the AzA yield was 70–80% [28].

An alternative to heavy metal oxidants was claimed by a Japanese patent in 2009, which reports the cleavage of methyl oleate in H_2_O_2_/H_2_O under subcritical conditions at 180–370 °C and 1–25 MPa. However, the reported yield was 31%, with concomitant drawbacks due to high-energy consumption and corrosion problems [28,33].

Two sustainable methods for the production of AzA as alternatives to the ozonolysis of oleic acid was reported by Benessere et al. The first method proceeds in two steps, coupling the oxidation of oleic acid (OA) to 9,10-dihydroxystearic acid (DSA) with oxidative cleavage by sodium hypochlorite. The second method involves a chemocatalytic system consisting of H_2_O_2_/H_2_WO_4_ for direct oxidative cleavage of the double bond of OA at 373 K [34].

A green route for the synthesis of azelaic and pelargonic acid starting from 9,10-dihydroxystearic acid (DSA) was reported by Kulik et al. by applying different supported gold catalysts in an aqueous solution. The reaction mechanism and the stability of the gold based catalyst were discussed in detail [35].

Recently, a green one-pot synthesis of azelaic acid and other valuable derivatives of oleic acid was proposed by Laurenza et. al. [36]. Rare earth metal (REM) triflates and commercial molybdenum dioxo dichloride (MoCl_2_O_2_) in the presence hydrogen peroxide allowed a selective oxidation of methyl oleate to azelaic acid or to methyl oleate epoxide.

A chemo-enzymatic route (Figure 1, route B) was described by Brenna et al. [2], which represents an alternative to ozonolysis for the transformation of oleic acid into AzA and PA in three steps. Initially, the peroleic acid is formed by epoxidation of the unsaturated acid (oleic acid) using a lipase in the presence of 35% H_2_O_2_. The resulting oxirane was subjected to in situ acid-catalyzed opening to form the diol. The obtained 9,10-dihydroxystearic acid was then chemically oxidized using atmospheric oxygen as a stoichiometric oxidant in the presence of catalytic quantities of Fe(NO_3_)_3_·9H_2_O, (2,2,6,6-tetramethylpiperidin-1-yl)oxyl (TEMPO) and NaCl, yielding the 9,10-dioxostearic acid. In the last step, the AzA and PA were achieved after the cleavage by 35% H_2_O_2_ under mild conditions in the absence of any other catalyst. The reported isolated yield for AzA was 44% and the purification did not require chromatographic methods.

Similarly, a Chinese patent reports an environmentally friendly technology for preparing AzA by an enzymatic catalysis oxidation system. The oleic acid undergoes the epoxidation catalyzed by nonspecified lipases in the presence of H_2_O_2_ and a noble metal, followed by the cleavage of the resulted diol to obtain AzA and PA. The reported AzA yield was 60–70% [37].

AzA can be synthesized also through fermentative oxidation with a 67% yield, as reported in a patent of Anderson et.al. The fermentation of oleic acid or triglycerides with Candida tropicalis yields 1,19-nonadec-9-enoic acid that is transformed by ozonolysis in the presence of Na-X zeolite catalyst. The advantage of this method, although the fermentative oxidation time takes up to 180 h, is that the formation of PA is avoided. The method was reported as useful for the oxidation of PA to AzA [38].

Only a few biocatalytic methods for AzA synthesis are reported in the literature (Figure 1A). Song et al. developed a multistep enzymatic procedure for the synthesis of AzA from oleic acid, 10-hydroxystearic acid [40] and vegetable oils [39]. The biocatalytic route consisted of the use of recombinant *Escherichia coli* cells expressing the genes encoding an oleate hydratase from *Stenotrophomonas maltophilia*, an alcohol dehydrogenase (ADH) from *Micrococcus luteus*, and a Bayer–Villiger monooxygenase (BVMO) from *Pseudomonas putida* KT2440 for the transformation of oleic acid into 9-(nonanoyloxy)nonanoic acid. In the further step, the hydrolysis of this compound was mediated by a cell extract of *E. coli* expressing the esterase gene from *P. fluorescens* to pelargonic acid and 9-hydroxynonanoic acid followed by the oxidation catalyzed by an ADH from *P. putida* GPo1. The reported substrates for AzA synthesis were olive oil, soybean oil, fatty acid methyl esters from microalgae and yeast derived oils. Although the described biocatalytic route is interesting, the concentration of the final product in the reaction medium did not exceed 5–10 millimolar.

Finally, a German patent described in 1974 the oxidation of the terminal -CH_3_ group of PA by *Debaryomyces pfaffii*, but the AzA yield was only 6% [41].

## 4. Azelaic Acid in the Synthesis of Bio-Based Polymers

In the last few decades several studies were focused on combining renewable and biodegradable polyester synthesis mainly driven by environmental reasons. In this context, different building blocks obtained from renewable resources have been proved as suitable raw materials for the synthesis of polymers [42].

In 2018, from the total amount of 2.11 million tons of bio-based plastics, about 57% (1.2 million tons) were nonbiodegradable [43]. Currently, modified starch-based bioplastic and several polyesters, including poly(lactic acid) (PLA), aliphatic-aromatic copolyesters, polyhydroxyalkanoates (PHAs), and poly(butylene succinate) (PBS) are the main biodegradable polymers available on the market [44].

The use of dicarboxylic acids as raw materials was studied by several groups, especially for the synthesis of polyesters due to the commercial potential of these products. Poly-(ethylene succinate) (PES) and poly(butylene succinate) (PBS) are promising materials for many conventional plastic replacements because of their biodegradability, acceptable mechanical strength, and comparable softening temperature to low-density polyethylene and polystyrene [45].

The enzymatic synthesis of polyesters starting from dicarboxylic acids (C4-C16) and different diols became a green alternative to harsh reaction conditions and, starting from the 1980s, lipases were successfully used as catalysts by different groups [46].

### 4.1. Azelaic Acid Based Copolyesters

Different polyesters of azelaic acid were reported up to date in the literature. The successfully used diols and dicarboxylic acid as co-monomers for the AzA polyesters are summarized in Table 1, divided onto bio-based and non-bio-based co-monomers. It can be observed that the enzymatic synthesis up to date was performed using three bio-based monomers: glycerol, pentaerythritol and PEG. Among the non-bio-based monomers, 1,6-HDO was used via the enzymatic route. All the other polyesters were synthesized via chemical catalysis. Details relating to the applications, properties and synthesis conditions are included in the following subchapters.

#### 4.1.1. Chemical Synthesis and Properties of Azelaic Acid Oligo and Polyesters

**Figure 2:**
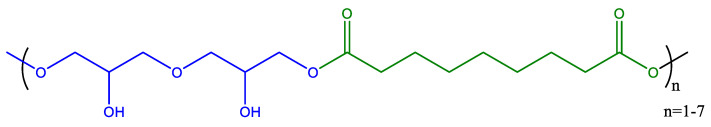
Chemical structure of the polyglycerol-azelaic acid polyesters obtained using diglycerol and different vegetable oils as raw materials.

The synthesis of the polyesters mentioned in Section 4.1 was performed in different reaction conditions and linear or hyperbranched products were obtained. For linear polymer/copolymers different diols and dicarboxylic acid were tested. The hyperbranched polyesters involved the use of glycerol as polyol together with free acid or the dimethyl ester of azelaic acid. The M_n_ determined value was in the range 1852–5410 g/mol and the highest value was reported for dibutyltin (IV) oxide as catalyst [48]. The synthesis of polyglycerol-azelaic acid polyesters (Figure 2) starting from polyglycerol-3/diglycerin and different vegetable oils in the presence of *p*-toluene sulfonic acid at different molar ratios and temperatures in the range 175–180 °C was patented by Giuliani et. al. The reaction products were analyzed by SEC and were tested for several cosmetic applications [57].

Glycerol-diacids oligoesters including polyglycerol azelate were synthesized by the same group using different catalysts such as titanium(IV) butoxide, [52,53] and [dibutyltin(IV) oxide] [51]. The reactions were performed at 150 °C in 22 h in a solvent-less system or in the presence of organic solvents (DMSO/DMF or toluene). MALDI-TOF MS, 1D and 2D NMR techniques were used for product characterization. The most efficient catalyst was Ti(OBu)_4_.

The poly(glycerol azelate) polyester was synthesized also at 125 °C in aluminum pans [49] or at 140 °C under nitrogen atmosphere [50]. The degradation of the products was also tested and the results indicated that the biodegradation upon burial in soil was slower than the hydrolysis after incubation in PBS buffer [50].

The synthesis, thermal characterization and enzymatic degradation of poly(ethylene azelate) was also reported. The polymers were synthesized by two-step melt polycondensation using Ti(OBu)_4_ as catalyst at 190 °C. GPC, DSC, WAXD and other techniques were used for characterization. The biodegradation of poly(ethylene azelate) was studied in PBS solution (chemical hydrolysis) and using a mixture of lipases from *R.delemar* and *P. cepacia*, indicating a faster degradation as compared to poly-ɛ-caprolactone [56]. 

Cho et al. obtained glycerol esters starting from six different dicarboxylic esters, including dimethyl azelate. Among the tested substrates, the highest conversion was obtained when dimethyl azelate was used, yielding 2,3-dihydroxy-propyl methyl azelate and 1,3-dimethoxyazelyloxy propan-2-ol [61]. Potassium hydroxide was used as catalyst and the mixture was heated at 80 °C. The obtained mono- and diesters were isolated and characterized by FT-IR, NMR, LC-MS techniques and their molecular weights were 277 g/mol and 461 g/mol.

The AzA-based polyesters synthesized by the chemical route were characterized by medium molecular weight determination and for some of them the thermal properties were evaluated. 

Among the reported data, the highest molecular weights were obtained for the terpolymers containing AzA, glycerol and a diol/diacid (Table 2). The melting point values in all cases were under 100 °C. For the terpolymers containing glutaric acid and diols as co-monomers, the melting point values were lower.

A series of copolymers containing AzA and glycerol have been reported by different groups (Table 2). The medium molecular weight values of most products were less than 10,000 g/mol except the reports of Wyatt et al. from 2012 when highest M_w_ value did not exceed 30,000 g/mol. No other thermal or mechanical properties of these polymers were reported.

All these results indicate that up to date the studies were focused on the catalyst development whereas minimal attention was devoted to structural analysis of the product.

#### 4.1.2. Technical, Cosmetic and Pharmaceutical Applications of Chemically Synthesized Polyesters Containing Azelaic Acid Moieties

Polymers containing AzA units were chemically synthesized by different groups and it was demonstrated that the insertion of AzA contributes to the flexibility, elasticity and hydrophobicity of the formed products [35]. Flexible polyester resins containing AzA are suitable for impact-resistant floor coverings. Furthermore, azelaic acid-based plasticizers are used to enhance low temperature flexibility in resilient polyvinyl chloride products [35] and more details will be discussed in the following section.

The patent of Giuliani et al, describes the incorporation of polyglycerol-azelaic acid polyesters into cosmetic compositions as active components at a concentration in the range of 0.01 to 20% (*w*/*w*). Formulations such as restructuring lotion, sebum-normalizing shampoo, tricological serum, conditioning cream, body milk, leave-on cleanser were prepared. The protective effect against thermal stress on the hair was demonstrated by treating locks with a shampoo and also with a leave-on formulation containing polyglyceryl-3-azelaiate with subsequent exposure to three consecutive heat cycles. 

Inflammation studies indicate that polyglyceryl-3-azelate has a significant inhibitory effect on TNF-α gene expression. Moreover, the polyglyceryl-3-azelate demonstrated an anti-inflammatory effect (reduction of TNF-α) greater than azelaic acid [57].

Azelaic acid diglycinate is presented in a patent as a bioconvertible compound since it is capable of releasing, after hydrolysis by lipase in the sebaceous follicle, at least one active agent against acne with antibacterial and/or anti-inflammatory activity [62].

### 4.2. Terpolymers Containing Azelaic Acid

**Figure 3:**
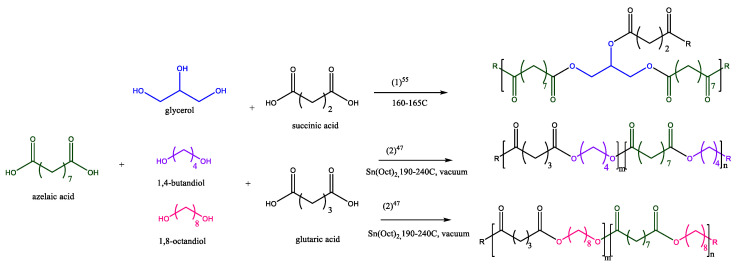
Polyesterification reaction of Aza, glycerol, succinic acid (1) [55]; synthesis of poly(butylene glutarate-co-butylene azelate) and poly(octylene glutarate-co-octylene azelate) copolyesters in two steps (2) [47].

For tuning the azelaic acid polyester properties, terpolymer synthesis was performed using different chain length diols or glycerol and aliphatic/aromatic diacids or correspondent diesters (Figure 3).

Baharu et al. synthesized new elastic polymers via polyesterification of glycerol with AzA and succinic acids. The polyesterifications were performed catalyst free at 160–165 °C for 2 h, followed by incubation in Petri dishes as films at 125 °C for 48 h. The chemical structure was proved by FT-IR, NMR analysis and the medium molecular weight was determined by SEC. The Ð values were in the range of 6.73–26.9 [55].

Poly(butylene glutarate-co-butylene azelate) (PBGA) and poly(octylene glutarate-*co*-octylene azelate) (POGA) copolyesters were synthesized by a two-step melt esterification at 190 °C and polycondensation process at 240 °C under vacuum using Sn(Oct)_2_. Different monomer compositions were tested and the products were characterized by GPC, NMR, TG, DSC, WAXD and tensile test. As expected, the isodimorphic structural behavior was affected by the chain length of the co-monomer diols [47].

Biodegradable aliphatic-aromatic polyesters including polybutylene terephthalate-butylenazelate were synthesized starting from dimethyl terephthalate, azelaic acid and a butandiol in a two-step process including a melt esterification at 200 °C and a polycondensation at 240 °C under vacuum [63].

### 4.3. Enzymatic Synthesis of Azelaic Acid Based Esters and Polyesters

Enzymes are efficient and sustainable alternatives to metal catalysts used in polycondensation (e.g., tin, titanium and antimony) since they are efficient at mild reaction temperatures ranging between 40 and 90 °C, whereas conventional chemo-catalytic polycondensations are carried out at T > 150 °C [64]. They also work in solvent-free systems and catalyze highly selective synthesis, enabling the production of functionalized polyesters with controlled architectures [65]. More importantly, enzymatically synthetized polyesters are inherently biodegradable since the same enzymes can catalyze both their synthesis and hydrolysis. Enzymatic polycondensation generally leads to products characterized by moderate molecular weight. According to Comerford et al., this drawback can be overcome by a two-step procedure where the biocatalyst is removed after a preliminary step, which is followed by a thermally driven elongation step [66]. Notably, polyesters with highly regular structures and molecular weights below 2500 g/mol have applications in cosmetic formulations in film forming [67] and are used for coating and adhesive applications.

Hydrolytic enzymes and lipases were employed for the synthesis of esters and polyesters of AzA, also with the aim of improving its compatibility, with respect to other ingredients of cosmetic and dermatologic formulation. It is known that AzA suffers from low-solubility, high melting point and large dosage requirement, which limit wide application in cosmetics and pharmaceutical products [58]. Moreover, some side effects are associated with the acid character of the molecule, demonstrated by high dosage pharmaceutical preparations.

**Figure 4:**
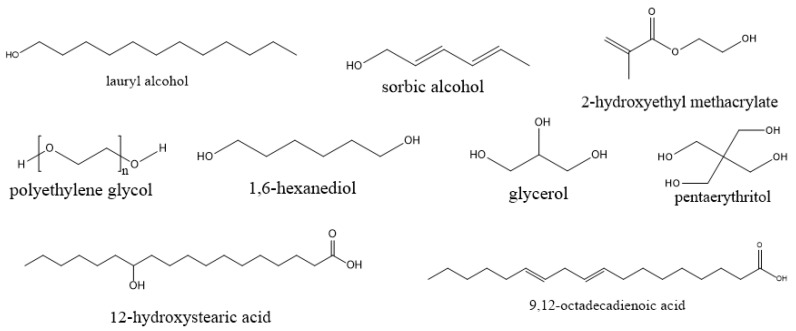
Typical examples of co-monomers used in enzyme-catalyzed polymerization of azelaic acid.

The enzymatic synthesis of esters and polyesters based on AzA are reported in few patents and research papers (Figure 4). In some patents related to the enzymatic synthesis of esters/polyesters starting from dicarboxylic acid and diols, AzA is mentioned as a possible reagent but no details related to the AzA synthesized and characterized products are given [68,69]. Khairudin et al. synthesized dilauryl azelate ester by using Novozyme 453 as catalyst. The synthesis was optimized in two reports, one by using an artificial neural network ANN-based design of experiment [58] and by central composite rotatable design [59]. The optimization of the process parameters included the enzyme amount, reaction time, reaction temperature, and molar ratio of substrates. Both methods proved their efficiency and high R^2^ (coefficient of determination) values were obtained.

Curia et al. synthesized AzA polyesters using 1,6-hexanediol and sorbic alcohol/12-hydroxystearic acid/trimethylolpropane oxetane/2-hydroxyethyl methacrylate as end capping molecule. The reactions were performed in scCO_2_ using Novozyme 435 as catalyst. The conversions were higher than 96% and the medium molecular weight were in the range of 1500–2400 [70].

In a later study, the same group synthesized specific end-functionalized amphiphilic copolymers based on azelaic acid, 1,6-hexanediol and PEG in scCO_2_. The M_n_ values determined based on GPC analysis were in the range 1700–3200 g/mol [60]. 

When dimethyl azelate was used as monomer together with glycerol in the presence of lipase Novozyme 435, a medium molecular weight of 2200 was obtained (400 mbar) in 48 h. When the pressure during the reaction decreased (150 mbar), a significant increase of the molecular weights (higher 20,000 g/mol) was observed [71]. Several lipases were mentioned in the summary of the invention, but the examples were performed only by using the commercial lipase Novozyme 435.

Highly branched high-molecular weight AzA polyesters were synthesized by a one-pot enzymatic system and reported by Nguyen et al. The first approach was focused on polyester synthesis starting from glycerol, azelaic acid, and tall oil fatty acid (TOFA) using Novozyme 435. The molecular weights after 25 h polymerization time, were in the range 20,900 to 39,700 g/mol with polydispersity indexes (Ð) between 3.2 and 5 (SEC analysis). The second azelaic acid derivatives was synthesized through a chemo-enzymatic reaction system. First, the pentaerythritol and azelaic acid were mixed and the reaction was performed at 180 °C and in the second stage the temperature was decreased and glycerol, azelaic acid, TOFA and Novozyme 435 were added. The SEC determined molecular weights were in the range of 16,800–57,800 g/mol and the Ð in the range 2.8–4.5 [54]. 

The enzymatically synthetized esters and polyesters of AzA were studied especially for their thermal properties (TG, DSC), their ability to form films, aggregates and for the cytotoxicity and antibacterial activity. The antibacterial activity of dilaurylazelate was evaluated against the pathogen bacteria *Staphylococcus epidermidis* S273 and the cytotoxicity was tested on 3T3 normal fibroblast cells. The results revealed that, compared to AzA, the ester is nontoxic, safe for pharmaceutical applications and presents promising antibacterial properties [59].

The thermal telechelics synthesized by Curia et al, starting from AzA, 1,6HDO and four different end-capping molecules, indicate that the T_m_ and the enthalpy of melting (∆H_m_) of the products are highly dependent on the end-capping molecule. It was clearly demonstrated that the bulkier structure of the end-cappers alters the crystalline structure [70]. 

Interesting properties were reported for copolymers based on azelaic acid, 1,6-hexanediol and PEG. Besides the thermal properties, the authors demonstrated that these polymers are capable of forming self-assembled aggregates in an aqueous environment. The thermal characterization of polymers revealed that a longer PHAz backbone presents larger crystallites (higher T_m_ and enthalpy of melting (ΔH_m_)). The aggregation in water was confirmed by comparison of NMR and the coumarin-6 loading tests proved the lipophilic molecules’ ability for dispersion and stabilization in an aqueous environment [60]. The products also showed significant surface tension reduction, indicating that the azelaic acid-based copolymers might find applications as surfactants in detergents and body-care formulations [60]. 

The AzA derivatives synthesized by Nguyen et al. were used for solid film preparation and characterized by DSC and water contact angle (WCA) measurements. The results showed that the acid/diacid composition had an effect for the hydrophilic/hydrophobic balance of the films. The AzA content was directly correlated to the increase of WCA up to 141 [54].

### 4.4. Polyamides Containing Azelaic Acid

**Figure 5:**
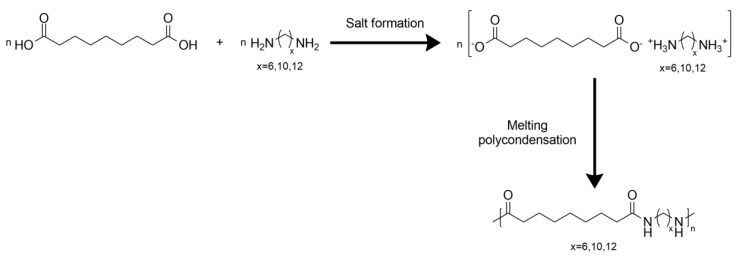
Synthesis and chemical structure of polyamide 6,9, polyamide 10,9 and polyamide 12,9.

Polyamides obtained by reacting AzA with different amines (Figure 5) are already commercial materials The most used is a polyamide synthetized from AzA and 1,6-diaminohexane, named nylon 6,9 (T_g_ 52 °C, M_w_ repeated unit 268 g/mol) [72].

Aliphatic polyamides, or nylons, are semicrystalline polymers with properties that change according to the ratio between amides and methylene groups. These materials are not biodegradable and possess great mechanical properties given by strong intermolecular hydrogen binding interactions [73]. Long-chain nylons, where the polar groups are separated by more than six carbon atoms, have both polyethylene properties and polyamide properties. They show low water absorption, good behavior at low temperatures, low density, and low hydrolysis sensitivity, the same as polyethylenic materials, and high melting temperature, good aesthetic properties and good processability, the same as polyamide materials [74].

Copolyamides of 1,4-butanediamine and a mixture of AzA and glutaric acid were synthetized with the aim of analyzing the structure of odd carbon number polyamides. The odd carbon number in their chain imposes a torsion of the amides groups to establish correct H-bonds, creating a γ structure. These polyamides presented a M_w_ in the range 40,000–51,000 g/mol, a predominant melting peak in the range 220–244 °C, T_d_ (thermal decomposition temperature) in the range 422–455 °C and a T_g_ in the range 50–71 °C. The higher M_w_ was achieved when AzA was 50% molar percentage in the acid mixture, while the lower T_g_ was obtained for the homopolymer with 100% AzA. Results suggested that a similar predominant hydrogen bonding structure was present in all the analyzed polyamides [73]. 

Tao et al. synthetized via step-melting polycondensation three different “environmentally friendly” aliphatic polyamides from AzA and 1,6-diaminohexane, 1,10-decanediamine or 1,12-diaminododecane, respectively [75]. AzA and 1,10-decanediammine are industrially obtained from plant oil and castor oil, respectively, while 1,6-diaminohexane can be obtained starting from biorenewable glucose and cellulose. They found that the diamine with a lower number of carbon atoms in its chain led to higher polymerization degrees, higher melting temperature and higher thermostability of the final material but higher T_g_, if compared to the ones with C10 and C12. With 1,6-diaminohexane, the final material had M_n_ 51,300 g/mol, T_m_ 214 °C, polymerization degree 190, T_d_ 435 °C and T_g_ 56 °C. With 1,10-decanediamine, the final material had M_n_ 38,900 g/mol, T_m_ 203 °C, polymerization degree 120, T_d_ 430 °C and T_g_ 52 °C. With 1,12-diaminododecane, the final material had number-average molecular weight 38,500 g/mol, T_m_ 195 °C, polymerization degree 100, T_d_ 425 °C and T_g_ 50 °C. A shorter chain in the diamino compound leads to the formation of more regular and symmetrical chains that are more easily polymerized and have a higher overall density of amide groups that increases the T_m_. Conversely, short chains give to the final material low flexibility and softness, and a decrease in T_g_ with the increase in CH_2_ group of the diamine cross-linker.

Interestingly, glycine addition to the nylon formulation confers biodegradability to the final product. When more than at least 2% *w*/*w* glycine is mixed with one polyamide-producing monomer, such as hexamethylene diamine with AzA, it is possible to obtain biodegradable nylon that can be used in devices such as gardeners’ tools, solving the environment plastic dispersion related to their use [76]. 

AzA polyamides with hot melt adhesive properties for PVC, steel, aluminum, wood, and textile materials are synthetized from different monomer mixture compositions. One composition example is AzA 41% equiv. polymeric fatty acids, 1,18-oxtodecane dicarboxylic acid and amines (ethylenediamine and piperazine) [77]. Another example is represented by dicarboxylic acid, including AzA, polymeric fatty acids, cyclic diamines or/and non-cyclic aliphatic diamines with an odd carbon number in their chain, and additionally organic diamines [78]. Copolymers with 0.5–25% *w*/*w* polyamine, with at least 11 N atoms, and equimolar quantities of dicarboxylic acids, including AzA, and eventually lactams or ω-aminodicarboxylic acids as ulterior polymer-forming monomers, show hot-melt adhesive properties [79]. The addition of AzA in the polyamide formulation of water-soluble textile adhesives, sizing agents or coatings renders them insoluble, and is therefore not recommended [80].

An electric conductive material with ferroelectricity was obtained through polycondensation of a diamine, composed of at least 50% mol of 2-methyl-1,5-pentanediamine, and a dicarboxylic acid mixture, composed of at least 50% mol of AzA. This polyamide is used as a sensor and noise-adsorbing material [81]. Moreover, a mixture of hexamethylene diamine with adipic acid, hexamethylene diamine with AzA and/or sebacic acid, and hexamethylene diamine with isophthalic acid and/or terephthalic acid was used to create transparent oxygen-barrier layers for food applications [82]. AzA is also found in a flame-retardant formulation composed of a thermoplastic polyamide resin, a thermoplastic polyester resin, a reinforcing or bulking filler and a fire retardant. AzA can be used as a component in both polyamide and polyester resins [83].

**Figure 6:**
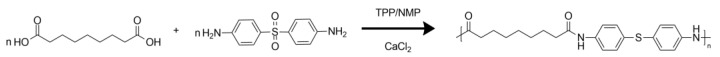
Polyamide synthesis from AzA and 4,4′-diamino diphenyl sulfone. TPP—triphenyl phosphite, NMP—N-methyl-2-pyrrolidone.

Finally, Modiri-Delshad et al. produced aromatic polyamide/amino acid Fe_3_O nanocomposites with increased thermal stability. Aromatic polyamides are classified as high-performance polymers for their high T_g_, good resistance to chemicals, temperature, and oxidation and they were obtained through the polycondensation of AzA, with 4,4′-diphenilsulfone (Figure 6), yielding products characterized by M_n_ 19,000 g/mol, M_w_ 42,000 g/mol and a main decomposition step at 420–470 °C [84].

## 5. Azelaic Acid as Component of Plasticizers and Lubricants

The first requisite for a plasticizer is to decrease the glass transition temperature (T_g_) of the final polymeric product, thereby enhancing its flexibility and workability [85]. In addition, both plasticizers and lubricants should have low volatility and low tendency for migration between surfaces to avoid changes over time in the finished product qualities and avoid environmental contamination [86].

Azelaic acid is widely used as plasticizer or lubricant in commercial products [87]. In the form of ester or polyester, it can be added to polymeric mixtures to vary the characteristics of the final product.

As a monoester, AzA esterified with aliphatic or aromatic alcohols, confers enhanced cold-temperature resistance to a vulcanizate of an olefinic copolymer. Dibenzyl azelate and diamyl azelate were added at 5–60% weight of the total composition as plasticizers in rubber-like polymers [88]. 

Different diesters of AZA were synthetized from natural precursors obtained through ozonolysis of crambe seed oil, which led to a mixture of brassylic and azelaic acid. These diacids, when reacted with alcohols of variable chain length at low temperature (4–10 °C) in the presence of a catalyst such as *p*-toluenesulfonic acid, yielded a mixture of diesters that were used at 32% in weight as plasticizers for PVC. Interestingly, the presence of AzA increases the PVC compatibility, the light stability, and the low-temperature flexibility, but also increases the tendencies to migrate outside the final product and the volatility [89]. 

Polyester plasticizers containing AzA gained importance during the 1970s as PVC plasticizers for their low volatility and low migration tendencies, and various patents dating back to that period will be referred to in the following. However, compared to simpler molecules, they had to be added in higher concentration to the final product, negatively affecting its mechanical properties, in particular at low temperature [90].

Hydroxypolyesters of AzA and 1,4-butanediol or 1,6-hexanediol with or without sebacic acid, were synthetized at high T (Table 3). Among the different molecules obtained, hydroxypoly-1,6-hexanediol azelate and hydroxypoly-1,4-butanediol azelate have a molecular weight about 40,000 g/mol and 25,000 g/mol respectively. These copolymers are used as PVC plasticizers. They are used in packaging and wrapping fields because of their low volatility and low tendency to creep, together with high cold impact resistance and good solvent extraction stability [91].

Polyester plasticizers based on dicarboxylic acids C4-C12, including AZA, glycols C2-C8 and 2,2-dibromomethyl-1,3-propane diol, show flame-retardant properties. These polyesters are added to synthetic resins, mainly foams, that have a high tendency to inflame in air [92]. 

To control the M_w_ of the product, AzA plasticizers are obtained with glycols and a terminator agent, such as hydroxyl and monocarboxy-substituted alkanes or mixtures of monobasic acids and monofunctional alcohols. The molecular weight of these polymers can be easily controlled by varying the glycols and the terminator chain lengths [93]. Other polyesters at low molecular weight were obtained reacting benzenedicarboxylic acids, dicarboxylic acids C5-C12 and neopentyl glycol-ethylene glycol mixture with a terminator agent, such as a monofunctional alcohol C6-C13 or a monocarboxylic acid C6-13. The dicarboxylic acids that showed the best results were AZA and adipic acids. The low Mw in the range 500–2000 g/mol confers to these polyesters a low tendency to migration and marring. These plasticizers have a high affinity for PVC resins, but low affinity for polystyrene and acrylonitrile butadiene styrene (ABS) resins, avoiding plasticizer migration between different plastic surfaces [94]. Differently, plasticizers compatible with different plastics such as PVC, rubber, PVC-like and rubber-like plastics were obtained starting from diacids, including AzA, with a mixture composed of 85–90% highly hindered diol and 10–15% short chain diol in the presence of catalysts [95].

New AzA-based plasticizers were created from PET recycling. AzA-based oligoesters were synthetized from the reaction of AzA with polyols at high T. These were then reacted with waste PET in the presence of a catalyst and 2-ethylenexanol as terminator. The plasticizer obtained can be used for PVC resins, substituting low M_w_ phthalates, which are dangerous for both the environment and human health [96].

**Table 3:** Structure, function and synthesis route of AzA-based lubricants and plasticizers.

Starting Material	Structure	Function	Synthesis Route	REF
AZA + 2-methyl-2-ethyl-1,3-propanediol	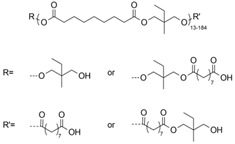	Lubricating oil	chem	[97]
AZA + nonanoic acid + trimethylolpropane (TMP)	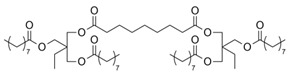	Lubricant	chem	[98]
AZA + propylene glycol mono-n-butyl ether	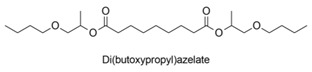	Lubricant diester	chem	[99]
diethyl azelate + 2-ethylhexanol	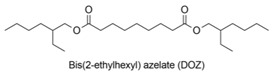	Lubricant	BIO	[100]
AZA + propylene glycol + lauric acid	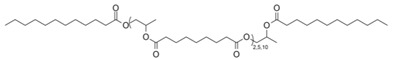	Plasticizer (PVC)	chem	[93]
AZA + 1,2-propylene glycol + lauric acid (terminator) + 2-ethylhexanol (terminator)	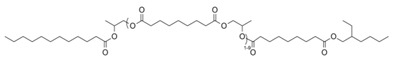	Plasticizer	chem	[80]
AZA + 1,4-butanediol	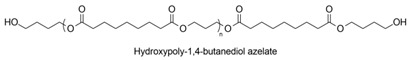	Plasticizer (PVC)	chem	[91]
AZA + alcohol C4-10 (n-butyl, isobutyl, n-pentyl, isopentyl, 2-methylpenthyl ecc)	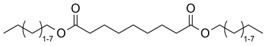	Plasticizer (PVC) diester	chem	[89]
AZA + benzyl alcohol	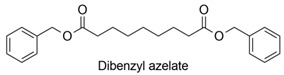	Plasticizer (rubber-like polymers)	chem	[88]
AZA + 1-pentanol	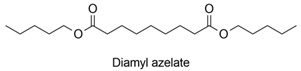	Plasticizer (rubber-like polymers)	chem	[88]

Similar to plasticizers, the requisites for lubricants are: a high viscosity index that means minimum viscosity change with temperature, a high flash point, good resistance to corrosion, high stability to oxidation, low temperature fluidity and low pour point, to reduce friction and facilitate movement between surfaces [101].

Bis(2-ethylhexyl) azelate, or DOZ, is a common commercial lubricant obtained from diethyl azelate and 2-ethylhexanol. DOZ has a M_w_ of 412.6 g/mol, a viscosity index of 138 and a pour point of −62.22 °C (PubChem). Recently, this diester was synthetized through the transesterification of diethyl azelate and 2-ethylhexanol in the presence of immobilized lipase B from *Candida antarctica* [100]. 

Di(butoxypropyl)azelate is synthetized from AzA and propylene glycol mono-n-butyl ether. Diesters and triesters of dicarboxylic acids with a carbon chain length of 9 or less, including AzA, and a branched monovalent glycol ether C3-C25 are good lubricant oils with excellent lubricity and low T characteristics. Notably, these lubricants have both low viscosity and low volatility because of their low Mw but high polarity that impedes their migration outside the final product [99]. 

Esterification of AzA with di-ethylene glycol and 2-ethylhexane in the presence of n-benzene disulfonic acid as catalyst, allows the production of two lubricants with a pour point of −53.89 °C and a flash point > 240.56 °C. It is possible to create lubricants “tailor made” from a dibasic acid C6-20, a glycol, and a mono-functional compound as terminator, in this case a mono-hydroxy alcohol or a mono-carboxylic alcohol. Varying the composition, polyesters with different lubricating properties, low pour point and flat viscosity–temperature properties were obtained [102].

One of the characteristics required for a lubricant is to have good oxidation stability. The presence of hydrogen in the β-position in the lubricant molecule is, therefore, a problem. To overcome this problem, hindered glycols with no hydrogen in the β-position were reacted with dicarboxylic acids, including AZA, in the presence of a catalyst. The obtained polyesters were oil-soluble and with a M_w_ in the range 10,000–1,000,000 g/mol. They are used as viscosity index improvers or thickening agents in lubricants, conferring to the final product a high viscosity index, enhanced shear stability and, most of all, good oxidative stability [103]. For the same reason, lubricants with a low pour point, made by reacting AzA with pelargonic acid and trimethylol propane (TMP) at 180–210 °C, were devised. The dicarboxylic and monocarboxylic acids can be obtained through ozonolysis of fatty acids from animal and vegetal sources, while TMP is a polyol with no hydrogen in the β-position [98]. Super-polyesters are created from AzA and 2-methyl-2-ethyl-1,3-propanediol. These polyesters have a M_w_ between 4000–50,000 g/mol and have good solubility in synthetic ester lubricating oils, working as thickening agents increasing viscosity, viscosity index and thermal decomposition resistance to the final lubricant [97].

## 6. Studies on the Biodegradation of Azelaic Acid Polymers

Azelaic acid, being a bio-based monomer, offers the opportunity to produce a more sustainable polymer as long as they meet some criteria associated with the efficient use of resources and more precisely: (i) the use of resources being cultivated on (at least) an annual basis; (ii) full valorization of biomass according to a cascade use; (iii) reduction of the carbon footprint and greenhouse emissions; (iv) saving and substituting fossil resources “step by step”. Consequently, the employment of AzA in plastic production may mitigate the “upstream” environmental impact of plastics, which refers to the impact generated from the extraction of raw materials to the manufacturing of plastic feedstock. However, the use of a bio-based monomer does not imply the automatic reduction of the “downstream” environmental impact of plastics, namely the impact generated once the consumer has discarded the product [104].

Indeed, polymers and plastics derived from biomass can be either biodegradable or non-biodegradable whereas there are different fossil-based plastics, such as polycaprolactone, that are biodegradable according to the relevant standards. On the other hand, there are several bio-based plastics on the market that are highly resistant to biodegradation due to their chemical structure (e.g., polyethylene from biomass). 

According to the United Nations Environment Programme (UNEP), 8 Mt of plastic are poured into the oceans each year, an equivalent to a full truckload every minute [105]. Collecting and recycling plastics represents an answer to the problem [106] but not all polymeric and plastic products can be collected and recycled, some examples include cosmetic ingredients, lubricants, food service plastics, mulching films for agriculture, and fishing nets. 

In the past, several methods such as thermal, pyrolytic, photochemical and photodegradation were used to solve the polyester environmental problem related to the end-of-life of packaging. However, the harsh degradation conditions, by-product formation and harmful gases are the major disadvantages that should be minimized, possibly through the biodegradation and recycling of organic carbon within biological pathways [107].

Ideally, the ecodesign of polymers and plastics should respond to the specific usage and disposal requirements of each different plastic product. Since biodegradation does not depend on the resource basis of a material, the misuse of bio-based plastics might lead to downstream environmental impact [30], which must be prevented through adequate and clear labelling. Because biodegradation occurs at different rates in soil and in water, there is the necessity for standards which define clearly how plastic waste must be managed in different environments. The European standard EN 13432 “Requirements for packaging recoverable through composting and biodegradation” [108] entails “at least 90% disintegration after twelve weeks, 90% biodegradation (CO_2_ evolvement) in six months, and includes tests on ecotoxicity and heavy metal content”. This is the standard for biodegradable packaging designed for treatment in industrial composting facilities and anaerobic digestion. Another standard, the ASTM D 6691 [109] offers a test method to assess biodegradation in water.

The mechanism of polyester biodegradation involves two major steps. First, a superficial degradation occurs due to the formation of a microbial biofilm generated after the hydrolysis of some ester bonds and small particles are generated. Then the enzymes secreted by microorganisms catalyze the depolymerization of the polymer chain into oligomers or monomers [110]. There are several factors that affect the enzymatic degradation of a polyester, such as its chemical structure, but also by their physical properties: crystallinity, melting point (T_m_), glass transition temperature (T_g_), etc. [111]. 

The following paragraphs report the up-to-date degradation methods applied to azelaic acid-based polyesters and polyesteramides. Among the degradation methods previously mentioned for the compounds containing azelaic acid, the studies were focused basically on enzymatic degradation, chemical degradation and biodegradation in sludge or compost. 

The enzymatic degradation of four different aliphatic polyester films containing 1,4-butanediol and dimethyl succinate, dimethyl glutarate, dimethyl suberate and dimethyl azelate units was evaluated by Shirahama et al. The experiments were performed at 37 °C and the enzymatic degradation of the polyester films were examined in buffer solutions using three different enzymes: cholesterol esterase from *Pseudomonas* sp., *R. delemar* lipase, and lipase B from *Pseudomonas fragi*. The experiments were performed at the optimal pH for each enzyme and the efficiency of the degradation was evaluated by mass loss measurement, molecular weight, and thermal properties. Among the tested enzymes the cholesterol esterase was the most efficient on the azelaic acid-1,4-butanediol polyesters. In the presence of the lipases, about 80% of the mass was lost in 200 h when lipase B from *Ps. fragi* was used, while the presence of the lipase from *R. delemar* was less effective (10% in 200 h) [112].

The chemical and enzymatic hydrolysis of poly(ethylene azelate) (PEAz) prepared by the two-stage melt polycondensation method was evaluated in comparison with polycaprolactone. The chemical hydrolysis rates, however, were very slow. The enzymatic hydrolysis was performed using a mixture of *R.delemar* and *P.cepacia* lipases at 30 °C in phosphate buffer pH 7. The degradation was monitored by weight loss measurement and even though compared to the PCL, the weight loss for PEAz was about four times higher, but since the molecular weight (PCL 60,000 g/mol, PEAz 21,000 g/mol), the melting point and the crystallinity of the samples were different, the authors indicated a comparable degradation rate with PCL. Morphological analysis by SEM confirmed the extension of the erosion surface in time and were correlated with the weight loss values [56].

In a later study, the same group synthesized and compared the enzymatic degradability of the poly(butylene azelate) (PBAz) polyesters with poly(ethylene azelate) (PEAz) and poly(propylene azelate) (PPAz). The samples were formulated as films and the enzymatic degradation studies were performed in similar conditions: 30 °C, phosphate buffer solution (pH 7.2) and a mixture of *Rhizopus delemar* lipase and *Pseudomonas cepacia* lipase (9:1 *w*/*w* lipases ration). The degree of biodegradation was estimated from the mass loss and were compared to the PCL enzymatic hydrolysis. The highest mass loss was observed for poly(propylene azelate) when after 35 days 40 mg/cm^2^ of the mass was lost while for the poly-ethylene and -butylene azelates the mass loss was significantly slower (less than 5 mg/cm^2^). The degradation behavior of the poly(propylene azelate) was attributed mainly to the lower crystallinity, 27% for PPAz, compared to 50–55% for PEAz and PBAz and 60% for PCL. Moreover, in the same study, a comparison of the mass loss values during degradation of the succinate, azelate, sebacate, polyesters and PCL was included. The results revealed that the enzymatic degradation rate of the azelaic acid polyesters was about four times lower compared to the succinic acid-based polyesters but twice as high, compared to the sebacic acid based polyesters. The results indicate that the enzymatic degradation in the presence of lipases is strongly affected by the samples’ crystallinity [113].

The enzymatic degradation of azelaic acid and 1,4-butanediol copolymers formulated as films was evaluated by using *Rhizopus oryzae* lipase at 25 and 37 °C up to 50 days, by weight loss monitoring and morphologically by SEM technique. The synthesis of the polyesters was performed by two-stage melt polycondensation at 150 °C and 180 °C. A comparison of the enzymatic degradability of polyesters containing azelaic and succinic acid moieties, as well as random copolymers containing 1,4-butanediol and the two dicarboxylic units, was performed and considerable differences were observed. The results indicated that copolymers have significant amorphous domains and facilitate enzymatic attack [114]. 

The degradation of a series of polyesteramides prepared from 1,4:3,6-dianhydro-D-glucitol α-amino acids and aliphatic dicarboxylic acids of the methylene chain length ranging from 2 to 10, including azelaic acid, was evaluated by three degradation methods: soil burial degradation, degradation in an activated sludge and enzymatic degradation. 

The soil burial degradation test was carried out at pH 6.8, temperature 27 °C, humidity 70–80% in the soil prepared at Nagoya University farm. The weight recovered after 60 days of incubation of the polyesteramide film containing azelaic acid was 45%. Compared to the polyesteramides containing a shorter methylene chain length, the recovered weight was higher.

The degradation in an activated sludge was performed at 27 °C and monitored by BOD and size exclusion chromatography measurements. The BOD biodegradability values obtained for the all the poly(ester amide)s series were in the range 0–40% while for the azelaic acid polyesteramides, the value was 5%. 

For the enzymatic degradation studies, seven different enzymes were considered: porcine pancreas lipase, porcine liver esterase, *Rhizopus delemar* lipase, *Rhizopus arrhizus* lipase, *Pseudomonas* sp. cholesterol esterase, *Pseudomonas* sp. lipase and *Streptomyces rochei* carboxylase. The experiments were performed at 37 °C for 24 h and the degradation was monitored by water-soluble total organic carbon (TOC) measurement. In addition to polyesteramides for this study, the corresponding polyesters were also considered as substrates. For the azelaic acid-based substrates, the results obtained in the presence of the porcine pancreas lipase and papain are presented and discussed. The results revealed that the enzymatic degradability of the polyesteramide compared to the polyester was about three times lower and, compared to the other polyesteramides from the series, the degradability was dependent on the methylene chain length of the dicarboxylic acid component [115]. 

The hydrolytic degradation of poly(glycerol-azelaic acid) synthesized via melt polycondensation at 140 °C in the absence of catalyst and solvent and of the correspondent hydroxyapatite nanocomposites was studied by Chenani et. al. Overall, the weight loss of the samples was gradually decreasing, which can be attributed to the interactions of water molecules with ester bonds in the PGAZ macromolecule. However, incorporating nanoparticles led to enhanced weight loss and hydrolytic degradation rate. The number of ester bonds has grown in the presence of higher amounts of nanoparticles and the water affinity and tendency improved. The hydrolytic degradation was evaluated in neutral conditions (pH 7) and in alkaline conditions (pH 11). In neutral conditions, the weight loss of the samples over 30 days was monitored and the gradually decrease of the samples in time was attributed to the interactions of water molecules with ester bonds but the incorporation of the nanoparticles led to strengthening the weight loss and hydrolytic degradation rate. At pH 11, a more accelerated decrease of the mass was observed, which was attributed to possible hydrolysis in the presence of the hydroxylic ions, but for the nanocomposites the hydrolysis rate was slowed and the weight fraction mass was about 30% higher compared to the sample without the nanoparticles [116].

The biodegradation of poly(sorbital azelate-*co*-sorbital citrate) polyester films was evaluated by Kesavan et al. at pH 7 in a phosphate cradle arrangement for up to 90h and 38.8% of the mass was lost. The results were compared with the data obtained for poly(mannitol glutarate-co-mannitol citrate) in similar conditions and even though the weight loss was slightly higher (42.8), a difference of 4% cannot be considered significant [117].

## 7. Conclusions and Future Perspectives

Bio-based polymers and plastics are objects of interest for their potential contribution to the resolution of the environmental impact caused by fossil-based plastics, because they promote the transition towards renewable raw materials. The environmental benefit is magnified when the bio-based polymers are also biodegradable and biocompostable, since they allow for the re-introduction of the organic carbon into the biogenic cycles, for instance, in the form of compost for the soil. Nowadays, the research in the field of the synthesis of bio-based polymers is mature for the delivery of new polymeric products and solutions, which are competitive in terms of performance beyond being sustainable. 

The aim of the present literature study was to provide a new perspective on the potential of azelaic acid as bio-based monomer for the synthesis of an array of products applicable in different fields, from packaging, cosmetic and pharmaceutical use. Apart from its pharmacological properties, azelaic acid, due to its double carboxylic groups, was successfully used for different oligo and polymer synthesis with applications as a plasticizer or as lubricants. Moreover, in recent years, the biodegradation of azelaic acid derivatives has been evaluated and promising results have been obtained. This renewal of interest towards azelaic acid materials was emphasized since the possibility of obtaining bio-based products was demonstrated. Moreover, the importance and potential of azelaic acid for industrial applications was demonstrated by its industrial synthesis on a large scale. 

Fully green synthesis routes of the azelaic acid derivatives (polyesters, polyamides) by selection of suitable catalysts, reaction media or solvent-less conditions, already allow the production of products for the cosmetic and pharma industries. Consequently, several interesting new materials containing azelaic acid will be obtained through polymerization using bi- or tri-component systems and will represent a renewable alternative to existing materials. In conclusion, the wealth of knowledge gathered in the last decades paves the way to the development of advanced technological solutions able to combine and valorize the chemical and pharmacological properties of this monomer, while benefiting from the sustainability and biodegradability of the new polymeric products.

## Figures and Tables

**Table 1:** Co-monomers used for synthesis of AzA based esters/polyesters.

	Co-Monomer(s)	Synthesis Route	Reference
Bio-based monomers	1,4-butadiol + glutaric acid	Chemical	[47]
	glycerol	Chemical and enzymatic	[48,49,50,51,52,53]
	Glycerol + FA	Enzymatic	[54]
	succinic acid + glycerol	Chemical	[55]
	pentaerythritol	Enzymatic	[54]
Non-bio-based	ethandiol	Chemical	[56]
	1,8ODO + glutaric acid	Chemical	[47]
	diglycerol	Chemical	[57]
	lauric alcohol	Enzymatic	[58,59]
	1,6-HDO + ECM * 1,6-HDO +/− PEG	Enzymatic	[60]

* ECM—end caper molecule: Sorbic alcohol/12-hydroxystearic acid/trimethylolpropane oxetane/2-hydroxyethyl methacrylate; FA—fatty acid.

**Table 2:** Thermal and mechanical properties of AzA-based polymers.

Co-monomer	M_n_ [g/mol]	M_w_ [g/mol]	T_g_ [°C]	T_m_ [°C]	Young Modulus [MPa]	Reference
Glutaric acid + 1,4BDO/ Glutaric acid + 1,8ODO	45,100–60,300 45,900–61,200	81,800–121,200 86,700–120,400	−7.2...+22.2 +25.1...+43.3	+20.0...+45.5 +47.1...+62.7	52.–287 60.5–362.6	[47]
Glycerol + succinic acid	515–3656	13,883–24,626	−24...−7	84.7...91.5	n.d.	[55]
Glycerol	2316	3010.8	n.d	n.d	n.d.	[52]
Glycerol	3245	5970	n.d	n.d	n.d.	[53]
Glycerol	11,690	28,056	n.d	n.d	n.d.	[51]
Glycerol	n.d.	n.d.	n.d.	~100	n.d.	[49]
Glycerol	1852–2873	3111–6981	n.d.	n.d.	0.98–11.1	[50]
Ethandiol	21,000	47,040	−60	62	n.d.	[56]

n.d.—not determined.
